# Development of an artificial intelligence algorithm for automated detection of cardiovascular intervention-related complications

**DOI:** 10.1007/s12471-026-02082-2

**Published:** 2026-10-21

**Authors:** Arnoud Okken, Rob Wanders, Andre Klappe, Kasper F. Beukema, Tom Oirbans, Peter G. Noordzij, Anouk van Lent, Saskia H. Haanemaaier, Jippe C. Balt

**Affiliations:** 1 Department of Cardiology, St. Antonius Hospital, Nieuwegein, The Netherlands; 2 Parnas, IT-Services and Consultancy, Groningen, The Netherlands; 3 Department of Anesthesiology and Intensive Care Medicine, St. Antonius Hospital, Nieuwegein, The Netherlands

**Keywords:** Artificial intelligence, Cardiovascular intervention, Complication registration, Electronic patient record, Natural language processing

## Abstract

**Background:**

Intervention-related complications negatively affect healthcare quality and costs. Complication registration and reporting play an important role in the improvement of healthcare outcomes but are time-consuming and hampered by under-reporting.

**Methods:**

We developed an artificial intelligence (AI)-aided tool that extracts specific complications, including bleeding, tamponade, CVA and phrenic paralysis, from electronic healthcare records of patients undergoing pulmonary vein isolation (PVI), percutaneous transluminal coronary angioplasty (PTCA) and transcatheter aortic valve implantation (TAVI). Both structured and unstructured data were used, along with additional signals identified through machine learning. The model was optimized to achieve high sensitivity.

**Results:**

Electronic records of 6,564 patients undergoing PVI, PTCA, and TAVI, performed at our hospital from 2017–2022, were used. The final model detected the various complications with a sensitivity between 94.4 and 100%, while specificity ranged from 99.4–100%. This resulted in positive predictive values ranging from 47.5–81%, with F‑scores varying between 0.64–0.87.

**Conclusion:**

We developed an AI-driven tool capable of automatically detecting complications following cardiac interventions from patient healthcare records, achieving a very high sensitivity. Our tool enables registration of complications while significantly reducing administrative burden on the healthcare providers and data managers. It provides valuable and high-quality input for improvement cycles, thereby enhancing patient safety and healthcare quality.

**Supplementary Information:**

The online version of this article (https://doi.org/10.1007/s12471-026-02082-2) contains supplementary material, which is available to authorized users.

## Introduction

A complication is defined as an unwanted and potentially harmful outcome of a medical treatment or procedure [1]. Complications can negatively impact the benefit/risk ratio of a (proposed) treatment, and are to be avoided both from a quality of care and health-economics perspective, since they can cause significant morbidity and cost. Complication registration and reporting are the cornerstone of improving the awareness of complications, reducing their incidence and improving quality of care [2]. Under-registration of complications is well known, and it hampers a well-functioning improvement cycle (or Plan-Do-Check-Act [PDCA] cycle) aimed at improving healthcare outcomes [3]. Reasons for under-registration of complications are time constraints, absence of a well-functioning registration system, and fear of legal repercussions [4].

Due to its ability to methodically search large databases and discern patterns, artificial intelligence (AI) has the potential to aid in the automatic detection (and ultimately predict) of complications.

**Fig. 1:**
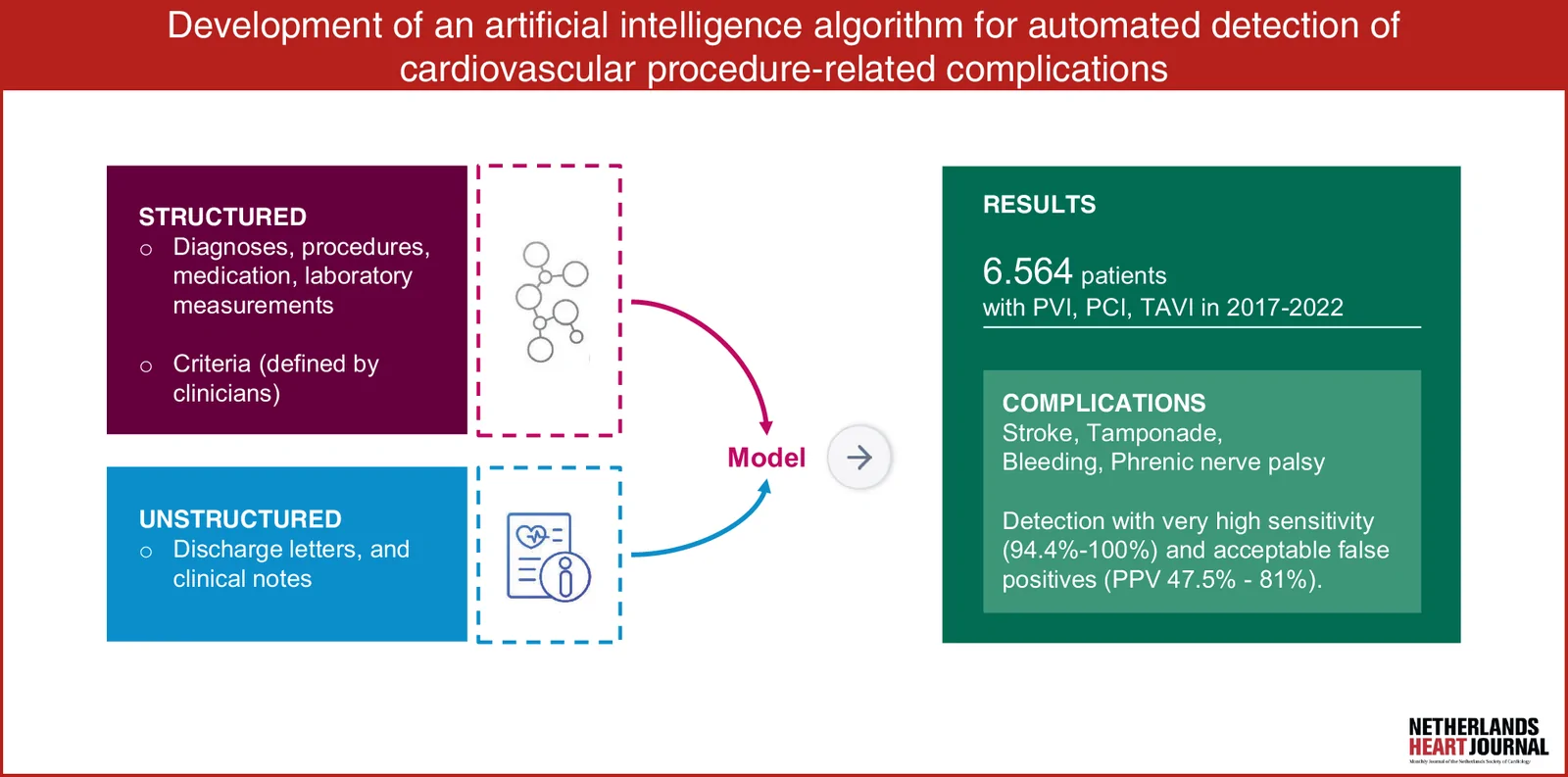
Infographic

In the present study, we describe the development of a fully automated, AI-driven complication registration system, independent of health care worker input, after percutaneous coronary intervention (PCI), pulmonary vein isolation (PVI) and transcatheter aortic valve implantation (TAVI) (Fig. 1). This algorithm was designed to identify key complications, including stroke, cardiac tamponade and vascular complications in Epic, an electronic patient record system (EPRS) that is used worldwide.

## Methods

We created an AI model designed to identify complications in EPIC healthcare files following cardiac interventions at the St. Antonius Hospital in the Netherlands. Following approval from the medical ethical committee, anonymized data were extracted from EPIC healthcare records, all analyses were performed on a compute cluster situated within St. Antonius Hospital. The dataset included all cardiac interventions (PVI, PTCA, TAVI) performed between 2017 and 2022 (*n* = 6,564), without additional inclusion or exclusion criteria beyond data availability. This approach was chosen to reflect routine clinical practice and to ensure representativeness of the target population. Multiple data sources were incorporated, such as the registered diagnosis, procedure logs, medication, laboratory measurements, clinical notes, and discharge letters. Predictors were extracted from all clinical records available during the index admission and up to 30 days after discharge, including data from readmissions and interdepartmental transfers. To ensure consistency, we adhered to data standardization using the OMOP Common Data Model and ZIB, a Dutch information standard mandatory for all hospitals. Standardization simplifies the integration of the AI model in current and future hospital settings.

The dataset was divided into a training and test set (*n* = 2,999) and a prospective validation set (*n* = 3,565). We developed models for specific complications—bleeding, tamponade, CVA and phrenic nerve palsy—defined by the Dutch Heart Registration (NHR; see Tab. 1). The definition for bleeding was expanded to include clinically relevant incidents, such as bleeding that prolonged hospital stay, requiring surgical intervention or blood transfusion. The primary objective was to achieve 100% sensitivity to avoid missing any complication, accepting a lower specificity and—consequently—false positives.

**Table 1:** Complications + Definitions

Complication	Definition
Stroke	Confirmed stroke or TIA caused by and within 72 h after a cardiac procedure
Tamponade	Confirmed or suspected pericardial effusion caused by and within 14 days after a cardiac procedure that requires intervention
Bleeding	Confirmed or suspected bleeding caused by and within 10 days after a cardiac procedure that requires intervention, transfusion or extends hospital stay
Phrenic nerve palsy	Confirmed or suspected phrenic nerve palsy caused by and within 14 days after a cardiac procedure

### Model development

**Fig. 2:**
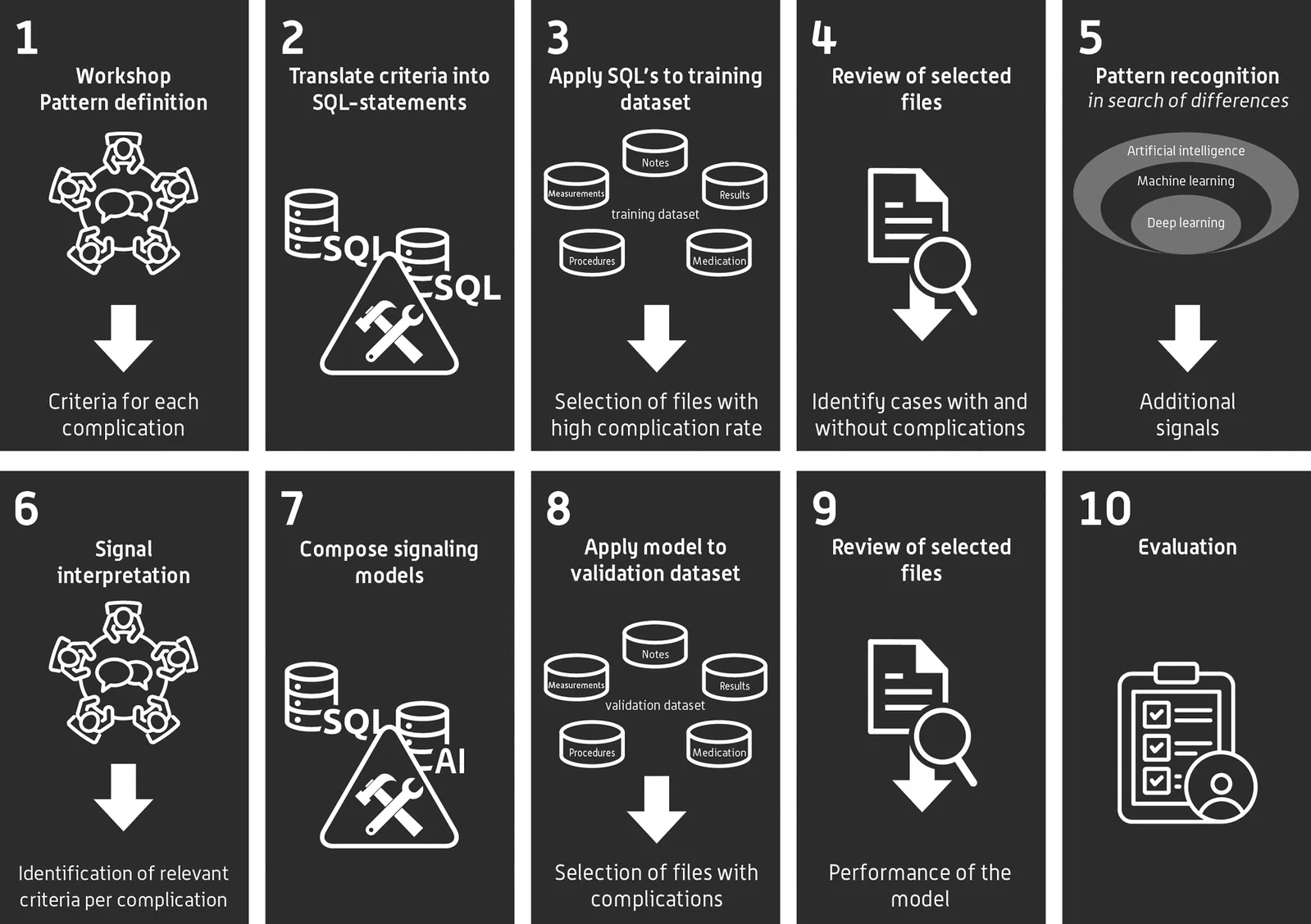
Ten Step Approach

We employed a systematic ten-step approach, outlined in Fig. 2.

*Steps 1–4* aimed to define clinical patterns and identify candidate cases in order to construct a dataset serving as a gold standard for each complication based on case review. Clinical presentations and patterns of each complication were discussed in workshops with healthcare professionals. Additionally, several patterns used to identify potential complications for submission to the Netherlands Heart Registry (NHR) were used. These patterns were translated into data statements, forming the basis for a weakly supervised dataset (see below for definition). Both structured data (medication, lab measures, data from procedure logs) and unstructured data (clinical notes and discharge letters) were used. Patients were flagged if any statement was met during an admission. Manual reviews of selected files (performed independently by authors KB and AO, with consensus adjudication involving AK for ambiguous cases) generated the final dataset used as a gold standard, which was used for model training and validation. No model predictions or outputs were available to the reviewers during the labeling process. A random sample of non-selected files, as well as a cross-check with existing registry data, did not identify additional complication cases.

*In Step 5*, machine learning was employed to identify additional signals in the training set.

*Step 6* involved assessing whether the identifiedpatterns were clinically plausible, removing spurious signals from the model. For example, the model identified administration of fluoroscopic contrast as an additional signal for CVA. This was removed because it correlated with the performance of a CT scan for the diagnosis of a CVA.

*Step 7* integrated the three models into one, combining the identified criteria from Step 2 using structured and unstructured data, together with the additional signals found with machine learning. Different models were applied for each complication, employing random forest (see below) and boosting classifiers (see below) for structured data and transformer embedding for unstructured data. Hyperparameter optimization was performed usingthe optuna framework (see next paragraph for high sensitivity). To enhance transparency and interpretability by incorporating explainable AI techniques were incorporated, including small decision trees and exploration of synonyms for common associated words with each complication.

*Step 8* tested the performance of the combined models on the validation dataset.

*In Step 9 and 10*, manual checks were conducted on all records labeled as complications together with a random sample of records without a complication. We are reporting specificity, sensitivity, and precision for each model on both the training and validation datasets by comparing the output of the models against the gold standard.

**Fig. 3:**
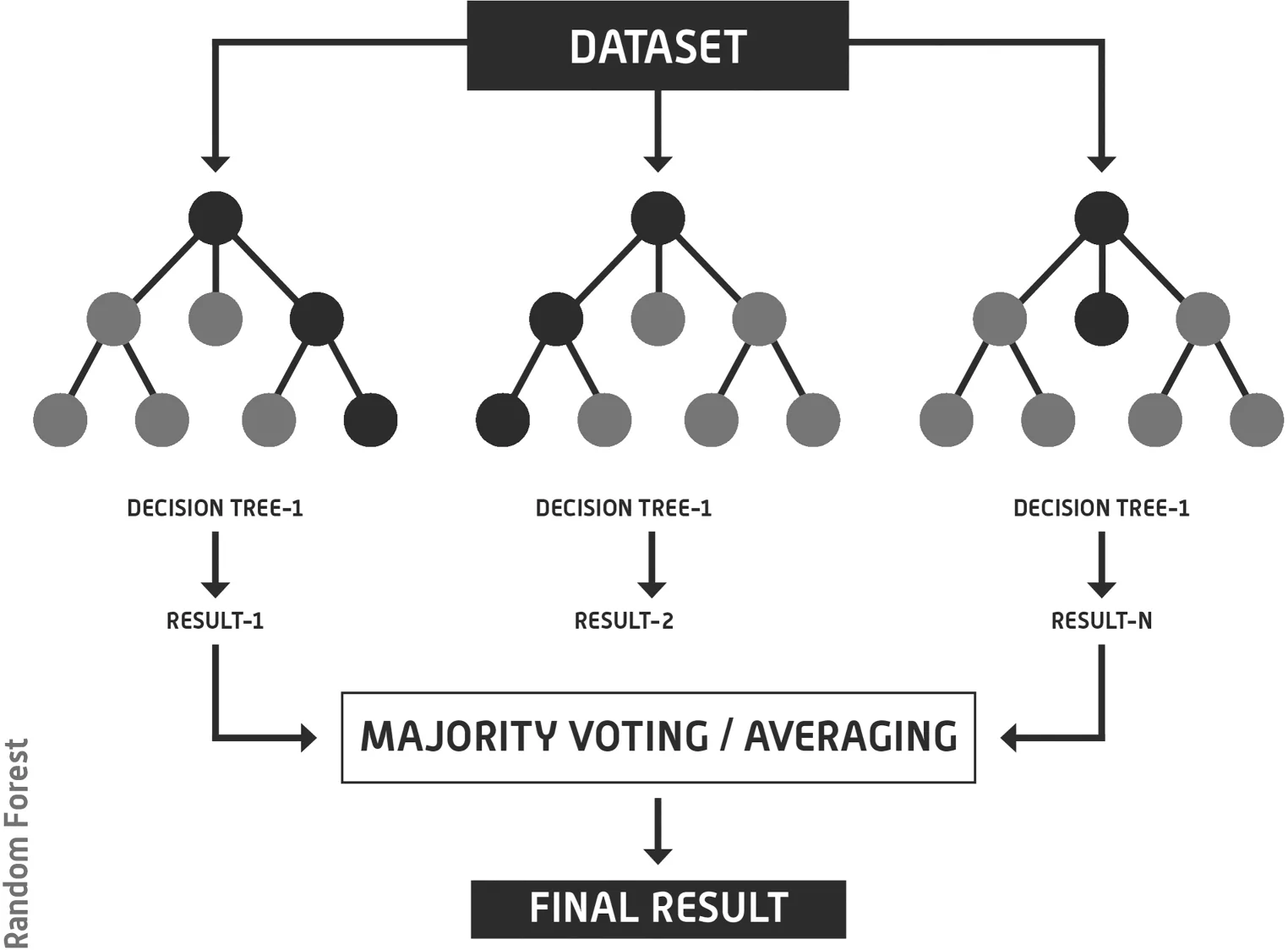
Random forest

For clarity, a short explanation of terms used can be found below:

- *Data statement*: A programming statement used to define and initialize variables or constants. They provide essential information about the characteristics of datasets.
- *Weakly labeled dataset*: A set of data that has not been explicitly labeled manually.
- *Random forest*: A machine learning algorithm that combines the predictions of multiple decision trees to generate a single output. (See Fig. 3)
- *Decision tree*: A type of supervised machine learning used to categorize or make predictions based on how a previous set of questions were answered. (See Fig. 3)
- *Boosting classifier*: A type of ensemble learning method in machine learning that combines multiple simple models to create a stronger, more accurate model.
- *Ensemble*: In the context of a boosting classifier, an ensemble refers to a collection of multiple weak learners. (typically decision trees)
- *OMOP Common Data Model:* Observational Medical Outcomes Partnership (OMOP) common data model is an open community data standard, designed to standardize the structure and content of observational data and to enable efficient analyses that can produce reliable evidence.
- *Transformer embedding*: A technique used in machine learning to convert words or tokens into vectors that capture the meaning and context. For instance, each word in a text is assigned a unique ID, and its position in the text and relation to other words (context) are put into vectors.
- *Optuna framework*: An open-source hyperparameter optimisation framework designed to automate the process of finding the best hyperparameters for machine learning models.
- *Hyperparameters* are pre-set configuration parameters that dictate how a machine learning model is trained. Unlike model parameters, which are learned from the data during training, hyperparameters are set by the practitioner.
- *Final dataset*: A set of data in which all complications have been labeled

### Statistical analysis

All statistical analyses were performed in Python. Models based on structured data were developed using scikit-learn (version 1.2.2). For unstructured data, transformer-based embeddings were generated using the Sentence-Transformers library with the all-MiniLM-L6-v2 model. Hyperparameter optimization was performed using the Optuna framework exclusively on the training dataset, optimizing the F‑score to prioritize sensitivity while maintaining acceptable specificity. In this real-world dataset, missing values were handled implicitly by the modeling approach without excluding records or applying explicit imputation techniques. For each complication, a final ensemble model was constructed by combining the predicted probabilities from the structured-data model, the unstructured-data model, and the criteria-based approach as input features. These inputs were subsequently combined using a logistic regression model and were trained using five-fold cross-validation to improve generalizability. Model development and validation were reported in accordance with the PROBAST-AI guidelines [5].

## Results

A total of 6,564 patients who underwent PVI, PCI or TAVI interventions at our hospital between 2017 and 2022 were included. Baseline characteristics are presented in Tab. 2. The dataset was split into a training and test set (*n* = 2,999) and a prospective validation set (*n* = 3,565).

**Table 2:** Patient characteristics

Age	67.0 ± 11.5
Body mass index	27.5 ± 5.8
Female sex	31.9%
Hypertension	42.0%
Diabetes mellitus	26.3%
Myocardial infarction	18.4%
Heart failure	20.4%
Atrial fibrillation	39.4%
Stroke	1.2%
Peripheral artery disease	5.1%

Tab. S1 (see Electronic Supplementary Material, ESM) presents the results of the initial case identification step (steps 1–4) applied to the training dataset. This screening step prioritized sensitivity to identify all potential complication cases for manual review, resulting in an intentionally high number of false positives.

The performance of the final ensemble models for detection of stroke, tamponade, bleeding, and phrenic nerve palsy after PVI, PTCA, and TAVI are presented in Tab. S2 (see ESM) and Tab. 3. A sensitivity between 94.4 and 100% was achieved, while specificity ranged from 99.4–100%. This resulted in positive predictive values ranging from 47.5–81%, with F‑scores varying between 0.64 and 0.87.

**Table 3:** Performance of the complication models—validation data

Complication	TP	FP	TN	FN	Sens	Spec	PPV	F‑score
Stroke	19	21	3525	0	100	99.4	47.5	0.64
Tamponade	24	18	3523	0	100	99.5	57.1	0.73
Bleeding	34	8	3521	2	94.4	99.8	81.0	0.87
Phrenic nerve palsy	2	1	3562	0	100	99.97	66.7	0.80

*TP* true positive, *FP* false positive, *TN* true negative, *FN* false negative, *SN* sensitivity, *SP* specificity, *PPV* positive predictive value

## Discussion

### Potential of AI in (cardiovascular) medicine

The use and potential of artificial intelligence in cardiovascular medicine are rapidly expanding. AI is being utilized and investigated in all aspects of cardiovascular medicine, such as diagnosis and risk stratification. For instance, AI-based software has demonstrated its ability to assist in the interpretation of ECGs [6]. It can support the quantification of coronary stenosis during invasive angiography [7] and optimize strategies for coronary revascularization [8]. In addition, it has shown excellent performance in cardiovascular risk stratification [9]. Previous studies have demonstrated the feasibility of using either unstructured text-based approaches [10] or structured data models [11] for the detection of complications. To the best of our knowledge, this is the first study to describe an AI-driven complication detection approach that integrates both structured and unstructured EHR data into highly sensitive models for the identification of cardiac complications.

### Complication registration for health care improvement

Recording and reporting complications form the foundation for increasing awareness, decreasing their occurrence, and elevating the quality of care [2]. Accurate complication registration can help physicians provide their patients and aid shared decision-making prior to procedures [12]. In addition, detailed knowledge of the causes, patterns and drivers of complications can be used for internal quality control. In other words, an accurate complication registration can drive a constant PDCA (Plan-Do-Check-Act) cycle, aimed at improving healthcare [13–15]. Also, improving Value-Based Health Care (VBHC) may be achieved by reducing its cost in terms of complications [15, 16].

However, under-registration of complications is widely recognized, posing a challenge to the effective functioning of a PDCA cycle designed to enhance healthcare outcomes [9]. The correct logging of complications in a designated section of the EPIC healthcare file was found to be no higher than 50% accurate at best, in our institution. To identify all complications, our data managers employ dedicated searches and manually review numerous patient records each year, a very time-consuming process.

### Performance of the algorithm

To minimize the risk of missed complications, maximizing sensitivity was a key objective during model development. The models for stroke, cardiac tamponade, and phrenic nerve palsy achieved a sensitivity of 100%, while the model for bleeding achieved a sensitivity of 94.4%. In addition, all models demonstrated excellent specificity performance, ranging from 99.4–100%. Despite success in achieving high sensitivity and specificity, there was an expected trade-off with a lower positive predictive value, as shown in Tab. S1 (see **ESM**). This is primarily due to the low incidence (2.2%) of complications in our database. Of note, when comparing our database to historical data, we observed similar rates of the aforementioned complications [17–19].

In practice, for a complication such as CVA, scrutinizing thousands of patient records, the algorithm will produce a shortlist of 40 records for manual review. This shortlist contained all 19 CVA cases, resulting in no missed event.

To facilitate comparison with previously published we calulated F‑scores for all models. (Tab. S1, ESM). These F‑scores range from 0.64–0.87, indicating an average to good level of performance [20]. While our aim was to achieve the highest sensitivity possible, this came at the expense of lower precision, consequently resulting in lower F‑scores. This is further demonstrated by the excellent performance of the AI model for bleeding, with an F‑score of 0.87, albeit with two false negative cases out of 36 total cases.

## Increase in efficiency

As mentioned before, the current method for identifying all complications in our department is an elaborate process that entails the manual review of numerous patient records each year. Reviewing all procedures performed annually for the presence of complications typically requires 100 h of work. Using the automated complication detection model to identify the actual 79 complications, only 127 patient records needed to be reviewed instead of over 6,500. Additionally, it is anticipated that the developed AI model will improve as additional data become available, given current advances in artificial intelligence.

## Limitations

Despite excellent results of the algorithm, there is room for improvement. For the complication of bleeding the algorithm missed two cases, resulting in a sensitivity of 94.4%. A challenge with this particular complication is its gradual scale, from a slight hematoma to a life-threatening bleed. If the algorithm is made too sensitive, it generates an excessive number of false-positive cases. On the other hand, if it is made less sensitive, it might miss cases.

Another challenge is the number of cases and complications. For a meaningful AI model, ideally large datasets, ideally comprising thousands of observations, are needed. As shown in Tab. S1 (see ESM), the total number of confirmed complication cases was small, particularly for phrenic nerve palsy, which was represented by only two cases. For instance, for the complication of phrenic nerve palsy, due to the limited number of patients with this complication, it was not feasible to develop a meaningful AI model for it. This model used selected criteria only, containing structured data (such as medication, lab measures, procedures) and unstructured data (such as notes and discharge letters). Future advances in artificial intelligence, including pre-trained large language models and zero- or few-shot classification approaches, may reduce the reliance on large labeled datasets for rare complications, although further validation will be required before clinical implementation. Finally, it was necessary to construct an AI model for every individual complication, a process that is time-consuming. Nevertheless, this development process only needs to be performed once.

### Implementation and future perspective

In our hospital, the automated detection algorithm is currently in use. It enables comprehensive registration and enhances patient safety and healthcare quality in a time-efficient manner [3, 4]. The total cost of implementing this project was approximately € 20,000–30,000, with an additional annual recurring cost of € 10,000. Despite not conducting an extensive cost-effective analysis, we believe it represents a modest investment that will yield significant benefits for patient safety and healthcare quality in the long term.

The ambition is to integrate our AI model into the electronic health record system, where it can autonomously document and display complications in a user-friendly way, accessible by all caregivers and mandated controllers. Furthermore, as the AI model gathers more data over time it might give insight into (obscured) risk factors for complications. Ultimately, the holy grail of automated complication detection is not only to identify complications after they occur, but also to alert healthcare providers to patients at high risk of developing complications, or even to predict complications before their onset. However, this objective remains beyond the scope of the current project.

## Conclusion

We developed an AI-driven tool capable of automatically detecting complications following cardiac interventions from patient healthcare records, achieving a very high sensitivity. The tool substantially reduces the administrative burden associated with complication registration while maintaining high-quality surveillance. It provides valuable and high-quality input for improvement cycles, thereby enhancing patient safety and optimizing healthcare quality.

### What’s new?

Procedure-related complications negatively affect healthcare quality and costs. Complication registration and reporting plays an important role in improvement of health care outcome but is time consuming and hampered by under-registration.

We developed an artificial intelligence (AI)-aided tool that extracts specific complications, including bleeding, tamponade, CVA and phrenic paralysis from electronic healthcare files of patients undergoing cardiac procedures with near 100% sensitivity and specificity.

The tool enables detection of complications while significantly reducing administrative burden on the health care providers and data managers. It provides valuable and high quality input for improvement cycles, thereby enhancing patient safety and healthcare quality.

## Supplementary Information

Table S1. Initial record selection for case identification based on steps 1–4—training data

Table S2. Performance of the complication models—training data

## Data Availability

All data supporting the findings of this work are included in the article.
