# Supplementary material for: Development of an artificial intelligence algorithm for automated detection of cardiovascular intervention-related complications
Source: Neth Heart J. 2026 Oct 21;34(11):407–13. doi: 10.1007/s12471-026-02082-2 (PMC13694613; doi:10.1007/s12471-026-02082-2)
Supplement: Supplementary file 1 [file 12471_2026_2082_MOESM1_ESM.docx]

Table S1. Performance of the model – training data

| Complication | TP | FP | TN | FN | SN^1^ | SP^1^ | PPV^1^ | F-score |
| --- | --- | --- | --- | --- | --- | --- | --- | --- |
| Stroke | 9 | 7 | 2983 | 0 | 100 (66.4-100.0) | 99.8 (99.5-99.9) | 56.3 (38.0-72.9) | 0.72 |
| Tamponade | 13 | 11 | 2985 | 0 | 100 (75.3-100.0) | 99.6 (99.3-99.8) | 54.2 (39.6-68.1) | 0.70 |
| Bleeding | 16 | 8 | 2975 | 0 | 100 (79.4-100.0) | 99.7 (99.5-99.9) | 66.7 (50.0-80.0) | 0.80 |
| Phrenic  nerve palsy | 2 | 2 | 2995 | 0 | 100 (15.8-100.0) | 99.9 (99.8-100.0) | 50.0 (20.0-80.0) | 0.67 |

^1^Values in parentheses represent 95% confidence intervals calculated using the exact binomial (Clopper–Pearson) method.
TP= true positive; FP= false positive; TN= true negative; FN= false negative; SN= sensitivity; SP= specificity; PPV= positive predictive value.
