# Supplementary material for: Development of an artificial intelligence algorithm for automated detection of cardiovascular intervention-related complications
Source: Neth Heart J. 2026 Oct 21;34(11):407–13. doi: 10.1007/s12471-026-02082-2 (PMC13694613; doi:10.1007/s12471-026-02082-2)
Supplement: Supplementary file 2 [file 12471_2026_2082_MOESM2_ESM.docx]

Table S2. Performance of the model – validation data

| Complication | TP | FP | TN | FN | Sens^1^ | Spec^1^ | PPV^1^ | F-score |
| --- | --- | --- | --- | --- | --- | --- | --- | --- |
| Stroke | 19 | 21 | 3525 | 0 | 100 (82.4-100.0) | 99.4 (99.1-99.6) | 47.5 (37.3-58.1) | 0.64 |
| Tamponade | 24 | 18 | 3523 | 0 | 100 (85.8-100.0) | 99.5 (99.2-99.7) | 57.1 (45.7-67.9) | 0.73 |
| Bleeding | 34 | 8 | 3521 | 2 | 94.4 (81.3-99.3) | 99.8 (99.6-99.9) | 81.0 (67.9-89.5) | 0.87 |
| Phrenic  nerve palsy | 2 | 1 | 3562 | 0 | 100 (15.8-100.0) | 99.97 (99.8-100.0) | 66.7 (22.0-93.4) | 0.80 |

^1^Values in parentheses represent 95% confidence intervals calculated using the exact binomial (Clopper–Pearson) method.
TP= true positive; FP= false positive; TN= true negative; FN= false negative; SN= sensitivity; SP= specificity; PPV= positive predictive value.
